# Effect of Empagliflozin on Thioacetamide-Induced Liver Injury in Rats: Role of AMPK/SIRT-1/HIF-1α Pathway in Halting Liver Fibrosis

**DOI:** 10.3390/antiox11112152

**Published:** 2022-10-30

**Authors:** Marwan A. ElBaset, Rana S. Salem, Fairouz Ayman, Nadeen Ayman, Nooran Shaban, Sherif M. Afifi, Tuba Esatbeyoglu, Mahmoud Abdelaziz, Zahraa S. Elalfy

**Affiliations:** 1 Pharmacology Department, Medical Research and Clinical Studies Institute, National Research Centre, 33 El-Bohouth St., Dokki, Cairo P.O. Box 12622, Egypt; 2 Pharmacology and Toxicology Department, Faculty of Pharmacy, October University for Modern Science and Arts, Cairo 12451, Egypt; 3 Pharmacognosy Department, Faculty of Pharmacy, University of Sadat City, Sadat City 32897, Egypt; 4 Department of Food Development and Food Quality, Institute of Food Science and Human Nutrition, Gottfried Wilhelm Leibniz University Hannover, Am Kleinen Felde 30, 30167 Hannover, Germany; 5 Pathology Department Medical Research and Clinical Studies Institute, National Research Centre, 33 El-Bohouth St., Dokki, Cairo P.O. Box 12622, Egypt

**Keywords:** antioxidant, inflammation, cytokines, hepatic fibrosis, caspase-3

## Abstract

Hepatic fibrosis causes severe morbidity and death. No viable treatment can repair fibrosis and protect the liver until now. We intended to discover the empagliflozin’s (EMPA) hepatoprotective efficacy in thioacetamide (TAA)-induced hepatotoxicity by targeting AMPK/SIRT-1 activity and reducing HIF-1α. Rats were treated orally with EMPA (3 or 6 mg/kg) with TAA (100 mg/kg, IP) thrice weekly for 6 weeks. EMPA in both doses retracted the serum GGT, ALT, AST, ammonia, triglycerides, total cholesterol, and increased serum albumin. At the same time, EMPA (3 or 6 mg/kg) replenished the hepatic content of GSH, ATP, AMP, AMPK, or SIRT-1 and mitigated the hepatic content of MDA, TNF-α, IL-6, NF-κB, or HIF-1α in a dose-dependent manner. Likewise, hepatic photomicrograph stained with hematoxylin and eosin or Masson trichrome stain of EMPA (3 or 6 mg/kg) revealed marked regression of the hepatotoxic effect of TAA with minimal injury. Similarly, in rats given EMPA (3 or 6 mg/kg), the immunohistochemically of hepatic photomicrograph revealed minimal stain of either α-SMA or caspase-3 compared to the TAA group. Therefore, we concluded that EMPA possessed an antifibrotic effect by targeting AMPK/SIRT-1 activity and inhibiting HIF-1α. The present study provided new insight into a novel treatment of liver fibrosis.

## 1. Introduction

Liver diseases are chronic and widespread worldwide. Hepatic fibrosis is a persistent toxic reaction associated with substantial morbidity and death. Chronic hepatic damage causes hepatic fibrosis. “Extracellular matrix” (ECM) buildup disturbs the natural construction of the liver, causing fibrosis. “Hepatic activated stellate cells” (HSC) are the first source of excess collagen [1]. After a fibrogenic stimulation, HSCs go from dormant to active Tumor necrosis factor-α (TNF-α), one of the cytokines released in inflammation that results in liver fibrosis by activating local HSCs and turning them into fibrogenic myofibroblasts [2]. Excessive ROS production, which leads to mitochondrial oxidative stress, apoptosis, and cell injury, is another pathway involved in liver fibrosis [3].

Thioacetamide (TAA), an organosulfur compound, has frequently been used to harm and induce fibrosis in the livers of experimental animals. As soon as TAA is given to rats, it breaks down into acetamide and thioacetamide-*S*-oxide. The metabolic intermediate of TAA, thioacetamide-S-oxide, attaches to certain macromolecules in the cell that are in charge of altering cell permeability and interrupting calcium storage. It also impedes mitochondrial function, which ultimately causes cellular destruction and the death of hepatocytes. TAA is a well-known liver toxin that causes hardly reversible fibrosis in rats that is similar to that in humans, making it an excellent model for testing prospective antifibrotic medications [4,5].

Sirtuins, a group of evolutionarily conserved class III histone deacetylases, depend on NAD. Sirtuin 1 (SIRT1), one of the seven members of the sirtuin family, has undergone the most research. They control a variety of cellular and physiological processes in both healthy and pathological states [6]. Moreover, SIRT1 has been implicated in the initiation and progression of a number of metabolic illnesses, such as alcoholic and non-alcoholic fatty liver disease (NAFLD) [7,8]. Additionally, SIRT1 demonstrates an inverse connection to NF-κB in regulating inflammation and is considered a master regulator of the inflammatory response in the liver [8]. Furthermore, higher production of α-smooth muscle actin (α-SMA) and collagen in fibrotic liver tissues is linked to SIRT-1 activation [9]. HIFs have been discovered as significant transcription factors that promote several facets of liver fibrosis, making them prospective treatment targets [10].

In individuals with type 2 diabetes, empagliflozin, an SGLT-2 inhibitor, lowers blood sugar via decreasing glucose reabsorption in renal tubules. Its mechanism of action is unique, as it is unaffected by pancreatic-cell activity or insulin sensitivity [11]. Empagliflozin has various favorable therapeutic effects in addition to its hypoglycemic effect, including uricosuric and natriuretic impact, as well as documented nephroprotective and cardioprotective properties [12]. Also, it has pleiotropic effects through diverse mechanisms of action, mediated via the AMPK-autophagy pathway, besides its role as an antioxidant [13]. Empagliflozin has been shown to improve liver steatosis and fibrosis in non-alcoholic fatty liver disease patients without diabetes [14]. Empagliflozin modulates both SIRT-1 in cardiac tissue [15] and HIFs in renal tissue [16]; however, its role in liver tissue has not been yet discovered.

The acceptable issue for hepatic dysfunction is good control for preventing disease progression leading to liver failure and cirrhosis. So, we aimed to demonstrate the biological activity of empagliflozin in the mitigation of TAA-induced hepatotoxicity via targeting SIRT-1 activity and inhibiting HIFs.

## 2. Materials and Methods

### 2.1. Animals

Forty adult male albino rats, weighing 180–200 g, were obtained from the Animal House at the National Research Centre (NRC, Cairo, Egypt). Rats were maintained under standard conditions of temperature (25 °C) with a 12 h (light)–12 h (dark) cycle. The animals were treated according to national and international ethics guidelines. All procedures and experiments have been approved (#Ph7/EC7/2021F) by the Research Ethics Committee-Faculty of Pharmacy, October University for Modern Sciences and Arts.

### 2.2. Chemicals

Thioacetamide (TAA, 98%) was obtained from Sigma-Aldrich (St. Louis, MO, USA). Empagliflozin (EMPA) was obtained from EVA Pharma (Cairo, Egypt). All compounds were of analytical grade.

### 2.3. Experimental Design

After an acclimatization period of one week, the following design was used for the four groups of rats, each with six rats:

Group 1: The control group: Rats were injected intraperitoneally with water containing 0.1% Tween 80 thrice weekly for six weeks.
Group 2: The TAA group: Rats were intraperitoneally injected with TAA (100 mg/kg, IP) thrice weekly for six weeks.
Groups 3 and 4: Rats received EMPA (3 and 6 mg/kg) [17] orally, daily for six weeks at the same time with TAA injection.

### 2.4. Body Weight Change

On the first and last days of the experiment, the body weight of each animal was recorded, and the following equation was used to determine the % change in body weight: %. 

Change in body weight (%): (body weight in the last day – body weight in the first day) × 100/body weight in the first day

### 2.5. Blood Glucose

Each rat had blood taken from its tail vein, and a portable Accu-Chek^®^ Active (Roche Diagnostics, Mannheim, Germany) was used to determine its blood glucose levels.

### 2.6. Blood and Tissue Samples

After 6 weeks, each rat had its blood drawn from the retro-orbital venous plexus while being lightly sedated with ketamine/xylazine. Sera were isolated from the blood samples which were drawn and stored at −20 °C for future biochemical studies. Animals were killed by cervical dislocation under anesthesia with IP ketamine-xylazine (K, 50 mg/kg; X, 10 mg/kg), and livers were quickly removed, cleaned in ice-cooled saline, blotted dry, and weighed. This procedure was carried out right after blood sampling. Rats from all groups had samples taken from the left lobe of their livers, which were then dissected and preserved in buffered neutral formalin at a 10% concentration for histopathological and immunohistochemical analyses. A second weighted component was stored in a freezer at −80 °C for future research.

### 2.7. Assessment of Serum Biochemical Analysis

Serum activities of aspartate aminotransferase (AST), total cholesterol, triglycerides, albumin, ammonia, and alanine aminotransferase (ALT) were estimated colorimetric procedure using commercial Biodiagnostic kits, Cairo, Egypt (Catalog No: AS 10 61 (45), CH 12 20, TR 20 30, AB 10 10, AM 1040 and AL 10 31 (45)), respectively. Similarly, gamma-glutamyl transferase (GGT) was determined by using a commercial BioVision Kit, Boston, USA (Catalog No: K784-100).

### 2.8. Determination of Tissue Protein

Protein content in the tissue was determined as per the guide of the protein estimation kit (Bangalore Genei, Bangalore, India (catalogue No.: 2624800021730)).

### 2.9. Preparation of Hepatic Tissue Homogenate

To create a 20% *w*/*v* homogenate, the later weighed portion of each hepatic tissue was pulverized with ice-cooled saline using a homogenizer (Medical Instruments, MPW-120, Warsaw, Poland). To eliminate cell debris, the homogenate was next centrifuged in a cooling centrifuge (Laborzentrifugen, 2k15, Sigma, Osterode am Harz, Germany) at 3000 rpm for 10 min at 4 °C. The aliquot was stored for further biochemical examination at −80 °C [18].

### 2.10. Assessment of Oxidative Stress Biomarkers

Liver-reduced glutathione content (GSH) and malondialdehyde (MDA) were determined colorimetrically as described by Abdel-Rahman et al. [19].

### 2.11. Assessment of Cell Regulatory and Inflammatory Markers

Liver contents of AMPK (Phosphorylated Adenosine Monophosphate Activated Protein Kinase), Adenosine Triphosphate (ATP), Nuclear factor-kappa B (NF-κB), Adenosine monophosphate (AMP), Sirtuin 1 (SIRT1), HIF-1α (Hypoxia Inducible Factor 1 Alpha) and Tumour necrosis factor-alpha (TNF-α) were estimated using ELISA kits according to the manufacturing instructions of Wuhan Fine Biotech Co., Ltd., Wuhan, China (Catalogue No.: ER0730), Cloud-Clone Corp, Katy, TX, USA (Catalogue No.: CEA349Ge, SEB824Ra), MyBioSource, San Diego, CA, USA (Catalogue No.: MBS7230212, MBS2023434), Elabscience, Houston, TX, USA (Catalogue No.: E-EL-R0513) and Abbexa, Cambridge, UK (Catalogue No.: abx050220), respectively.

### 2.12. Histological Examination

Liver specimens collected from various groups were fixed for 24 h in 10% buffered neutral formalin. Next, the fixed specimens were subjected to routine processing to obtain paraffin blocks. The later blocks were sectioned serially into 4–5 μm thick sections to be evaluated histologically and immunohistochemically. Sections from each group were stained with hematoxylin and eosin (H&E) and Masson trichrome stain [20,21].

### 2.13. Immunohistochemical Examination

According to the procedure outlined by Hsu et al., avidin-biotin-peroxidase and diaminobenzidine tetrahydrochloride DAB, Sigma-Aldrich (St. Louis, MO, USA) was used in immunohistochemical experiments to identify caspase-3 and α-SMA expression on paraffin slices of the control liver and all treatment groups [22]. For the avidin-biotin-peroxidase technique of detecting the antigen-antibody complex, tissue slices were incubated with a monoclonal antibody for caspase-3 and α-SMA (Dako Corp, Carpenteria, CA, USA) and the necessary chemicals (Vactastain ABC peroxidase kit, Vector Laboratories, Newark, USA). Using DAB, each marker expression was seen.

### 2.14. qRT-PCR Analysis for IL-6 Expression in the Liver Tissues

The Hepatic tissues from all different groups were homogenized. Total RNA was extracted with Direct-zol RNA Miniprep Plus, Zymo Research Corp, Irvine, CA, USA (Catalogue No.: R2072); quantity and quality of the RNA were assessed by a dual spectrophotometer (DU-800, Brea, CA, USA).

SuperScript IV One-Step RT-PCR kit, Thermo Fisher Scientific, Massachusetts, USA (Catalogue No.: 12594100) was utilized for reverse transcription of extracted RNA followed by PCR. A 96-well plate StepOne instrument (Applied Biosystem, CA, USA) was used in a thermal profile as follows: 10 min at 45 °C for reverse transcription, 2 min at 98 °C for RT inactivation and initial denaturation by 40 cycles of 10 s at 98 °C, 10 s at 55 °C and 30 s at 72 °C for the amplification step. After the RT-PCR run, the data were expressed in Cycle threshold (Ct) for the target and housekeeping genes. Normalization for variation in the expression of each target gene, IL-6, was performed by referring to the mean critical threshold (CT) expression value of the GAPDH housekeeping gene by the ΔΔCt method. The relative quantitation (RQ) of each target gene is quantified according to the calculation of the 2-∆∆Ct method. Primer sequence was; forward 5′-ATTGTATGAACAGCGATGATGCA-3′ and reverse 5′-CCAGGTAGAAACGCAACTCCAGA-3′ (gene bank accession number is NM_012589.2) for IL-6 gene, GAPDH housekeeping gene was forward 5′-CCTCGTCTCATAGACAAGATGGT-3′ and reverse 5′-GGGTAGAGTCATACTGGAACATG-3′ (gene bank accession number is NM_001394060.2).

### 2.15. Statistical Analysis

Before proceeding with the statistical analysis, data values were checked for normality using the Shapiro test. The data are presented as means ± S.E. Data were processed by one-way ANOVA followed by the Tukey–Kramer Post hoc test. GraphPad Prism software (version 9, USA) was employed to perform the statistical analysis and establish the represented graphs. The significance level was set to *p* < 0.05 for all statistical tests.

## 3. Results

### 3.1. Effect of Empagliflozin on the Percentage Change in Body Weight in Thioacetamide-Intoxicated Rats

**Figure 1:**
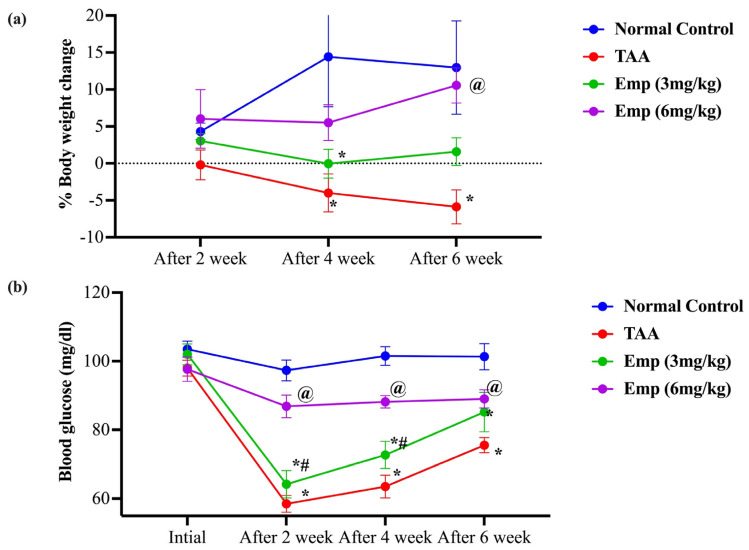
(**a**) Effect of empagliflozin on the percentage change in body weight in thioacetamide-intoxicated rats. (**b**) Effect of empagliflozin on blood glucose in thioacetamide-intoxicated rats. Data are presented as mean ± SE. * vs. normal control group, ^@^ vs. TAA group, ^#^ vs. Emp (3 mg/kg) at *p* < 0.05.

After two weeks of TAA injection, there was no observed body weight change between groups. However, after 4 weeks, the TAA and EMPA (3 or 6 mg/kg) groups suffered from a significant drop in percentage change in body weight by 128, 100, and 62% compared to the body weight change of the normal group, respectively. Similarly, after 6 weeks, only the percentage change in body weight of the TAA group continued dropping down by 145% and 88%, compared to the body weight change of the normal group, respectively. At the same time, only the EMPA (6 mg/kg) elevated the percentage change in body weight by 1.8-folds compared to the TAA group (Figure 1).

### 3.2. Effect of Empagliflozin on Blood Glucose in Thioacetamide-Intoxicated Rats

After two weeks of the study, the TAA lowered the blood glucose level of the TAA and EMPA (3 or 6 mg/kg) groups by 40, and 34% compared to the normal group, respectively. Likewise, the diminutions in the blood glucose were evident after 4 weeks by 37 and 28% and after 6 weeks by 25 and 16%, compared to the normal group, respectively. Noteworthy, only the EMPA (6 mg/kg) group showed comparable blood glucose level values to the normal group and was significantly higher than the TAA group after 2, 4, and 6 weeks by 48, 39, and 18%, respectively (Figure 1).

### 3.3. Effect of Empagliflozin on Serum Hepatic Function in Thioacetamide-Intoxicated Rats

Serum Albumin levels declined by around 0.395-fold in the thioacetamide group compared to the normal control rats. Moreover, rats given TAA + EMPA (3 or 6 mg/kg) showed an elevation of serum Albumin by 33.65% and 119.2% compared to the TAA group, respectively. At the same time, serum GGT levels were elevated tremendously by nearly 9.0-fold in the thioacetamide group compared to the normal control rats. Furthermore, rats given TAA + EMPA (3 or 6 mg/kg) showed a significant reduction of serum GGT by 46.5% and 77.8% compared to the TAA group, respectively. Similarly, the serum ALT levels were elevated tremendously by almost 5-fold in the thioacetamide group compared to the normal control rats. However, rats given TAA + EMPA (3 or 6 mg/kg) showed a significant reduction of serum ALT by 46% or 68% compared to the TAA group, respectively. Likewise, the TAA group indicated a 3.5-fold increase in serum AST level compared to the normal control group. On the other hand, TAA-intoxicated rats administered EMPA (3 or 6 mg/kg) showed a decline in AST level by 44% or 60% compared to the TAA group, respectively. Also, the rats of the TAA group presented a high amount of ammonia serum by 4.5-fold compared to the normal control group. On the other hand, rats of the EMPA (3 mg/kg) group reflected a lower percentage of 40.8%, while the EMPA (6 mg/kg) group showed the lowest percentage of ammonia by 68.5% than the TAA group (Table 1).

### 3.4. Effect of Empagliflozin on Lipid Profile in Thioacetamide-Intoxicated Rats

Triglycerides level in the TAA group was elevated by 2.2-fold compared to the normal group. However, the group of EMPA (3 or 6 mg/kg) showed lower triglyceride levels by 15% to 46%, compared to the TAA group. Similarly, the TAA group revealed a significant elevation of total cholesterol level by 2.8-fold compared to the normal group. Notably, EMPA (3 or 6 mg/kg) groups presented a drop in total cholesterol levels by 30% and 60% compared to the TAA group (Table 2).

### 3.5. Effect of Empagliflozin on Oxidative Stress Biomarkers in Thioacetamide-Intoxicated Rats

The hepatic glutathione (GSH) content was slightly decreased by almost 0.28-fold in the thioacetamide group compared to the normal group. However, rats given TAA + EMPA (3 or 6 mg/kg) showed a significant increase in hepatic GSH content by 79.3% and 241.37%, compared to the TAA group, respectively. Also, hepatic ATP contents were marginally decreased by almost 0.25-fold in the thioacetamide group compared to the normal control rats. On the other hand, rats given EMPA (3 or 6 mg/kg) presented a significant increase of hepatic ATP by 92.5% and by about 2.5-fold, compared to the TAA group, respectively. Similarly, the hepatic AMP content was partially reduced by nearly 0.33-fold in the TAA group compared to the normal group. Furthermore, rats given EMPA (3 or 6 mg/kg) showed an enormous increase of hepatic AMP by 92% and by about two-fold, compared to the TAA group, respectively. On the contrary, the hepatic malondialdehyde (MDA) content in the TAA group exhibited an approximately 5.6-fold increase compared to the normal group. Moreover, rats that received EMPA (3 or 6 mg/kg) revealed a significant decline of serum MDA by 35.08% and 75.06% compared to the TAA group, respectively (Table 2).

### 3.6. Effect of Empagliflozin on Hepatic Inflammatory Biomarkers in Thioacetamide-Intoxicated Rats

**Figure 2:**
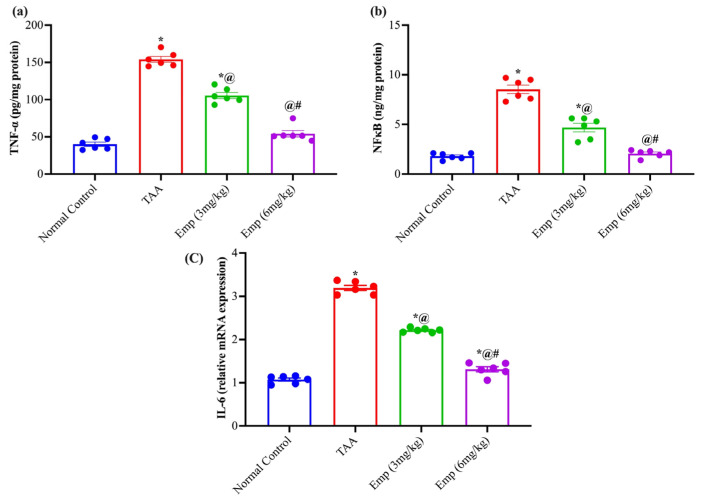
Effect of empagliflozin on hepatic (**a**) TNFα, (**b**) NFκB, and (**c**) IL-6 relative expression in thioacetamide-intoxicated rats. Each bar represents as mean ± SE. * vs. normal control group, ^@^ vs. TAA group, ^#^ vs. Emp (3 mg/kg) at *p* < 0.05.

The hepatic tumor necrosis factor-alpha (TNF-α) content in the thioacetamide group was significantly elevated by nearly 3.8-fold compared to the normal group. On the other hand, the administration of EMPA (3 or 6 mg/kg) to TAA-intoxicated rats dropped the hepatic TNF-α content by 31.5% and 64.8% compared to the TAA group, respectively. At the same time, the hepatic nuclear factor-kappa B (NF-κB) content of the TAA group was significantly elevated by nearly 4.7-fold compared to the normal group. Noteworthy, rats treated with EMPA (3 or 6 mg/kg) showed lower contents of NF-κB by 45.0% and 75.7% than the TAA group. Relatedly, the hepatic IL-6 expression in the TAA group showed an approximately 3-fold increase compared to normal control rats. Additionally, rats given EMPA (3 or 6 mg/kg) disclosed a significant drop in hepatic IL-6 expression by 30.5% and 58.9% compared to the TAA group, respectively (Figure 2).

### 3.7. Effect of Empagliflozin on Hepatic AMP-Activated Protein Kinase, HIF-1α, and Sirtuin-1 in Thioacetamide-Intoxicated Rats

**Figure 3:**
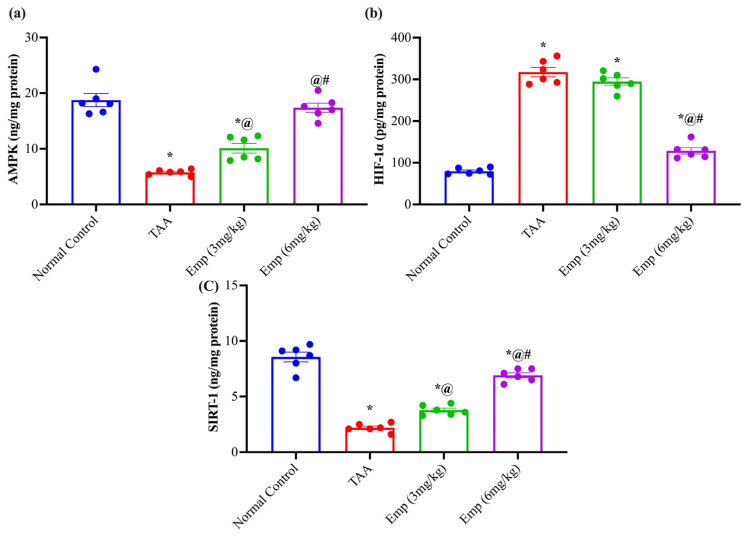
Effect of empagliflozin on hepatic (**a**) AMP-activated protein kinase, (**b**) HIF-1α, and (**c**) Sirtuin-1 in thioacetamide-intoxicated rats. Each bar represents mean ± SE. * vs. normal control group, ^@^ vs. TAA group, ^#^ vs. Emp (3 mg/kg) at *p* < 0.05.

The TAA group experienced a significant depletion of AMP-activated protein kinase (AMPK) content by 0.30-fold compared to the normal group. While, the treatment with EMPA (3 or 6 mg/kg) replenished the AMPK content by about 1.6- and 3-fold compared to the TAA group, respectively. At the same time, the rats of TAA displayed higher contents of Hypoxia-inducible factor 1-alpha (HIF-1α) by approximately 3.9-fold compared to the normal group. Nonetheless, the administration of EMPA (3 or 6 mg/kg) to TAA-intoxicated rats reduced the hepatic content of HIF-1α by 7 and 59% compared to the TAA group, respectively. Together, the Sirtuin-1 (SIRT-1) content was greatly diminished by 74.3% in the TAA group compared to the normal group. Remarkably, the administration of EMPA (3 or 6 mg/kg) to TAA-intoxicated rats restored the SIRT-1 content by 1.7- and 3.1-fold compared to the TAA group, respectively (Figure 3).

### 3.8. Histological Examination

**Figure 4:**
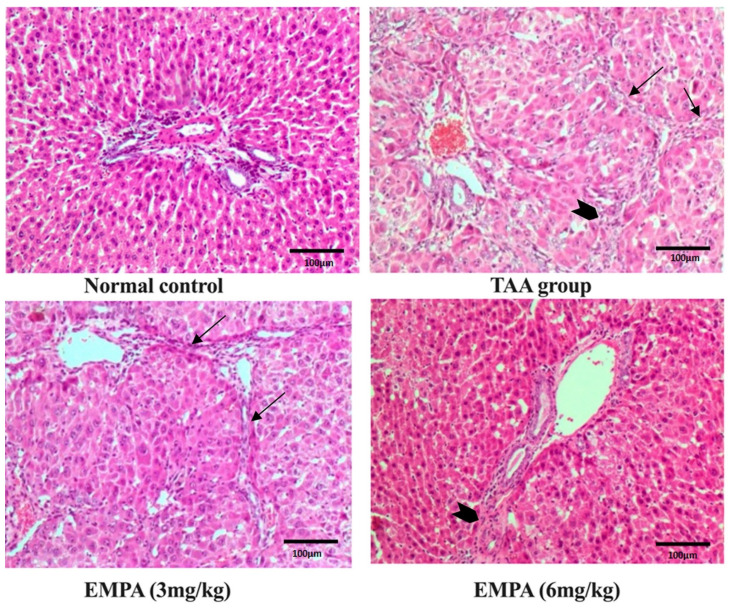
Photomicrographs of histopathology sections stained with hematoxylin and eosin. Normal histology of the control group, moderate inflammation (arrow head) and marked fibrotic septa with complete cirrhotic nodule formation (thin arrows) in the toxic group, marked regression in inflammatory cell infiltrate however, minimal regression of fibrosis (thin arrows) in 3 mg group, excellent regression of both inflammatory cell infiltrate and fibrotic process in 6 mg group (arrow head) (magnification = 200×).

**Figure 5:**
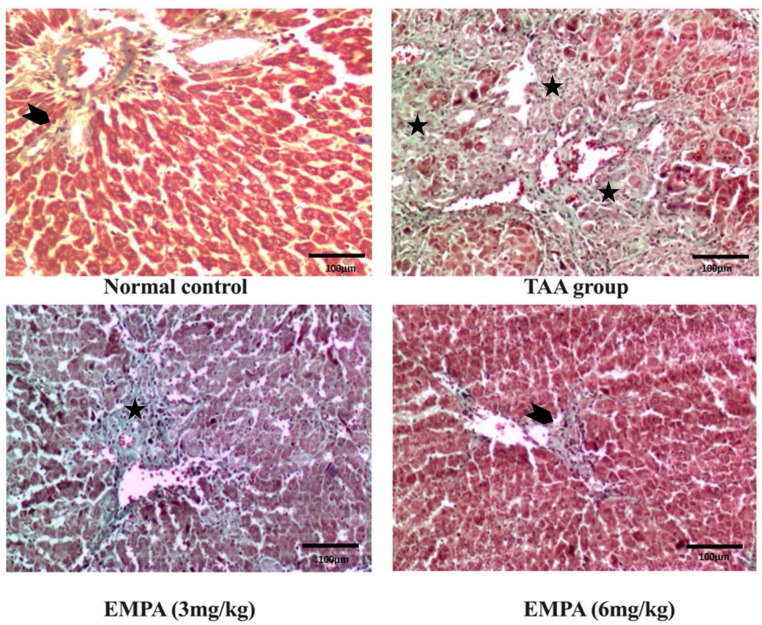
Photomicrographs of histopathology sections stained with Masson trichrome. The control group with only thin collagen in the wall of normal vascular structures (arrow head), disturbed architecture, and marked fibrosis in the toxic group (three asterisks), same findings as the toxic group in the 3 mg group (black asterisk), and regression of bands with minimal fibrosis (arrow head) in the group that received 6 mg. (magnification = 200×).

Sections from the liver tissue revealed marked hepatotoxicity in the form of marked fibrosis and cirrhosis in the group that received TAA with moderate inflammatory cell infiltrate compared to liver sections from the normal group stained with hematoxylin and eosin (H&E) or Masson trichrome stain. At the same time, sections from liver tissue of the group that received EMPA (3 mg/kg) revealed regression of inflammatory cell infiltrate; however, minimal deterioration of the hepatotoxic fibrotic effect of TAA with marked fibrosis and complete nodules formation (cirrhosis). The Portal tract showed attempted regeneration with moderate portal tract proliferation. Also, a regression of inflammation is detected with periportal and central mild inflammation compared to the group that received TAA in contrast to liver sections from the TAA group stained with hematoxylin and eosin (H&E) or Masson trichrome stain. Together, the liver tissue of the group that received EMPA (6 mg/kg) revealed marked regression of the hepatotoxic effect of TAA with minimal to no fibrosis and minimal inflammation simulating the density accepted in the control group, in comparison to the TAA group stained with hematoxylin and eosin (H&E) or Masson trichrome stain (Figure 4 and Figure 5).

### 3.9. Immunohistochemical Examination

**Figure 6:**
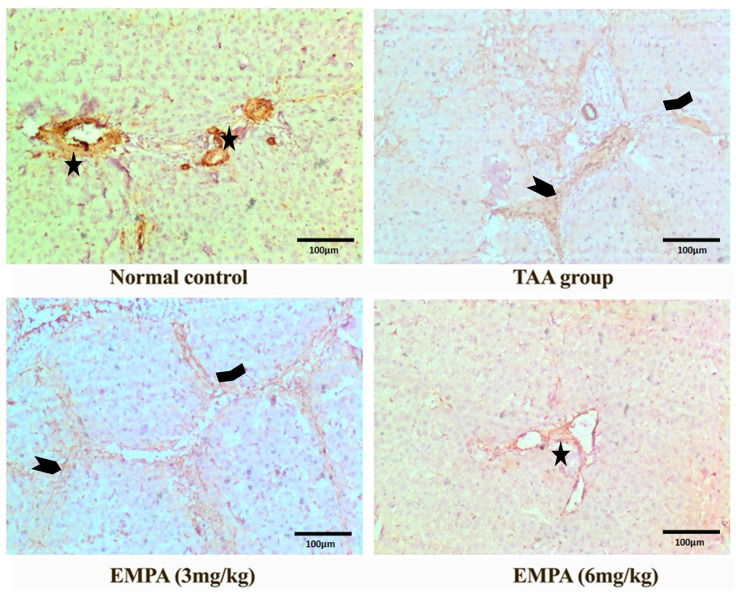
Photomicrographs of histopathology sections stained with immunohistochemical actin antibody. Only positive fibers in the wall of vascular structures (Asterisk) with no abnormal fibers deposition in the control group, positive thick fibers in a periportal fashion with porto-central (wide arrow head) and porto-portal extension (arrow heads) in the toxic group, persisting positive thick fibers and nodular cirrhotic pattern (arrow heads) with 3 mg group, regression of stain with almost negative expression in 6 mg group (magnification = 200×).

**Figure 7:**
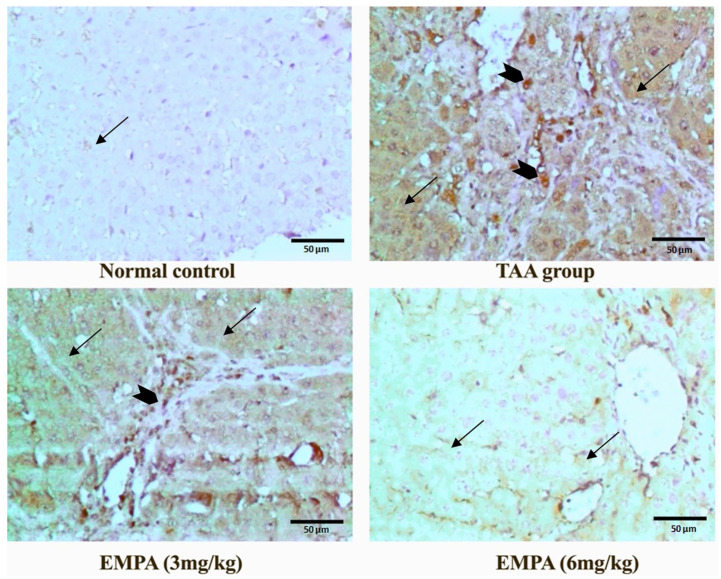
Photomicrographs of histopathology sections stained with immunohistochemical caspase-3 antibody. Control group was minimally stained mainly sinusoidal (thin arrow), with strong diffuse cytoplasmic stain (thin arrows) in hepatocytes with occasional nuclear staining in the portal area (arrow heads) in the toxic group; insignificant changes in comparison to the toxin group with strong diffuse hepatocyte cytoplasmic (thin arrows) and occasional nuclear staining (arrow head) in the portal area in 3 mg group; minimal positive staining restricted to the sinusoidal surface (thin arrows), no hepatocyte expression in 6 mg group (magnification = 400×).

The photomicrograph of the normal group revealed positive fibers in the wall of vascular structures with no abnormal fibers deposition of the immunohistochemical stain for smooth muscle actin antibodies (Figure 6). Similarly, the immunohistochemical stain for caspase-3 revealed minimal stain, mainly sinusoidal in the normal group (Figure 7). On the contrary, the photomicrograph of the TAA group disclosed positive thick fibers in a periportal fashion with the porto-portal and porto-portal extension of the immunohistochemical stain for smooth muscle actin antibodies (Figure 6). In a similar way, the Immunohistochemical stain for caspase-3 exposed a strong diffuse cytoplasmic stain in hepatocytes with occasional nuclear staining in the portal area of the TAA group (Figure 7). Nonetheless, the photomicrograph revealed persisting positive thick fibers and nodular cirrhotic pattern of the immunohistochemical stain for smooth muscle actin antibodies (Figure 6). In a similar way, the Immunohistochemical stain for caspase-3 displayed an insignificant change in comparison to the toxin group with strong diffuse hepatocyte cytoplasmic stain and occasional nuclear staining in the portal area (Figure 7). Interestingly, the photomicrograph of the EMPA (6 mg/kg) portrayed comparable staining to the normal group for either the smooth muscle actin stain or the caspase-3 (Figure 6 and Figure 7).

## 4. Discussion

Liver cirrhosis is the 14th most common cause of death, responsible for over a million deaths per year worldwide. Survival significantly decreases when the disease progresses to a decompensated phase [23]. Unfortunately, there is no clear therapeutic option until now that could curb or treat the progression of liver fibrosis and its consequences. For this reason, we decided to explore the possible protective role of empagliflozin (EMPA) in curbing the progression of liver fibrosis in thioacetamide (TAA)-intoxicated rats via targeting AMP-activated protein kinase (AMPK)/Sirtuin-1 (SIRT-1) activity and via inhibiting Hypoxia-inducible factor 1-alpha (HIF-1α). TAA is a well-known hepatotoxin that causes fibrosis in rats nearly irreversible and identical to human fibrosis, making it a good model for testing prospective antifibrotic medicines [4,24]. In the current study, the administration of TAA thrice weekly to rats induced an enormous elevation in serum ALT, AST, GGT, and ammonia, accompanied by a sharp decline in serum albumin. The rise of non-plasma liver enzyme activities following TAA administration implies their leakage of hepatocytes in response to injury with toxins affecting the integrity and function of liver cells. Also, it signifies an injurious marker for liver damage [4,25]. Furthermore, TAA-induced hyperammonemia is a metabolic change marked by elevated amounts of ammonia, a nitrogen-based molecule that influences the brain’s cognitive function [26]. Besides the TAA-induced hypo-albuminemia is a consequence that leads to impaired hepatocellular function accompanied by ascites, that in turn may develop renal failure associated with a further increase in mortality [27]. This milieu is attributed to the rapid conversion of TAA into TAA-S-oxide after being given to rats, which can bind to large molecules and induce cell permeability and conductivity deterioration by affecting calcium reserves. It also reduces mitochondrial activity, which leads to cellular destruction and hepatocyte death [28,29]. The current investigation found that EMPA can correct this derangement in serum parameters and halt hepatocyte structure and function deterioration. This finding is well-kept in previous studies in which EMPA reduced liver enzymes in the hepatic steatosis model preclinically [30,31] and clinically [14,32].

The TAA-intoxicated rats suffered from hepatomegaly, demonstrated by the increase in liver/weight ratio compared to the normal group. This setting could be explained in light of the progression of liver fibrosis as documented by Al-Attar and Shawush [33]. Interestingly, EMPA preserved the relative liver weight in a dose-dependent manner, according to the previous results on the mouse model of non-alcoholic steatohepatitis [31].

The TAA-induced oxidative stress was evidenced by the increase of malondialdehyde (MDA) and adenosine monophosphate (AMP) along with a decline in the hepatic content of both glutathione (GSH) and adenosine triphosphate (ATP) in the TAA group. TAA’s toxicity is caused by its bioactivation into reactive metabolites in the liver, which results in the generation of reactive oxygen species (ROS) that cause oxidative stress. GSH depletion, a decrease in SH-thiol groups, and oxidation of cell macromolecules, including lipids, occur due to these processes. GSH is essential for detoxifying ROS and reactive electrophilic chemicals [34]. The generation of reactive ROS is linked to an increase in lipoperoxidation. GSH must be depleted below a threshold level to produce a significant rise in lipid peroxidation and cell damage. There are two primary GSH pools in cells: cytosolic (more than 80%) and mitochondrial [35,36]. Mitochondria are the primary biological generator of ROS. Mitochondrial GSH depletion can result in mitochondrial oxidative stress and ROS release from the mitochondrial matrix into the cytoplasm [37]. Besides, ROS triggered an impediment in the mitochondria leading to decreased ATP and increased levels of AMP. This instability leads to impairment in the AMPK, the critical metabolic sensor and regulator [34,38]. Remarkably, EMPA retracted the hepatic MDA and ATP decline by ameliorating GSH and AMP levels dose-dependently. The current results agreed with Lee et al., in which the hepatic ROS content of fatty murine was hampered, and antioxidant enzymes were upregulated via the c-Jun N-terminal kinase pathway [39].

The TAA administration upsurged the TAA group’s serum triglycerides and total cholesterol levels. TAA is processed via the mixed-function oxidase system and lipid peroxidation, altering the lipid profile attributed to the impairment of hepatocyte function. The result was in line with a previous study on TAA-induced hyperlipidemia [40]. In contrast, EMPA protracted the rise in lipid profile to comparable levels with the normal control group. Jojima et al. reported the anti-inflammatory and anti-steatotic of EMPA in streptozotocin-indued steatohepatitis [31]. Similarly, Perakakis and his colleagues document the beneficial effect of EMPA on improving metabolic and hepatic derangement in mice models of non-alcoholic fatty liver disease (NAFLD) [32]. Likewise, Nasiri-Ansari and his coworker showed that EMPA mitigated a high-fat diet’s deleterious effects on the lipid profile in liver cells of ApoE(-/-) mice via autophagy and halting oxidative stress [30]. Furthermore, a previous clinical trial confirmed the hepatoprotective effect of EMPA in nondiabetic subjects with NAFLD and hepatic fibrosis [14].

TAA resulted in substantial increases in hepatic contents of IL-6 and TNF-α, as well as activated form NFκB in the current study, which is consistent with the findings of Ali et al. [41]. Furthermore, our results were reinforced by the histopathological findings. TAA-induced hepatic fibrosis was evident in the hematoxylin and eosin and the staining with Masson trichrome stain with marked complete cirrhotic nodule formation and fibrosis collagenous connective tissue fibers. Additionally, the TAA group was intensely stained with immunohistochemistry of caspase-3 and actin stain, empathizing the deleterious effect of TAA-induced fibrosis. The liver is thought to be an essential organ in regulating cytokine activity and synthesis, which is influenced by the early pro-inflammatory cytokines produced by macrophages. TAA, as a hepatotoxin, induces hepatic macrophages (Kupffer cells) to produce pro-inflammatory and inflammatory cytokines and mediators, such as TNF-, IL-6, and NFκB, which have crucial roles in hepatic inflammation [20]. Furthermore, this setting leads to the activation of caspase-3, an apoptotic marker. The apoptotic pathway cascade comprises fourteen caspases [42]. Caspase-3 is one of the most crucial execution proteases among them. Caspase-3 is a sensitive marker for liver injury that has also been linked to hepatic fibrosis [43]. The increased expression of caspase-3 in TAA-induced rats suggests that the inflammation caused by TAA led to hepatocyte apoptosis in the latter stages [20]. Moreover, Eissa et al. and El-Mihi et al. suggested that TAA-induced liver fibrosis is associated with the elevated expression of caspase-3 and expression of alpha smooth muscle actin; this agreed with our current outcomes [44,45].

EMPA mitigated the hepatic content of TNF-α, IL-6, and NFκB in a dose-dependent manner that aligns with previous in vitro and in vivo investigations, in which EMPA lessens hepatic steatosis [46]. Furthermore, EMPA restored the TAA-induced derangement in hepatic ATP and AMP that in turn influenced the replenished hepatic AMPK content. Previous research by Lee et al. revealed that AMPK activation suppressed the activation of cultured hepatic stellate cells and macrophages and decreased thioacetamide-induced liver fibrosis in mice, aligning with reduction in the expression of alpha smooth muscle actin. In particular, AMPK regulates sirtuins in cooperation with another physiological detector, the NAD^+^-dependent deacetylase, to modulate gene expression. Apoptosis, cellular senescence, endocrine signaling, glucose homeostasis, aging, lifespan, and mitochondrial biogenesis are just a few of the biological processes that sirtuins regulate. Sirtuin 1 (SIRT1), in particular, controls a variety of processes during oxidative stress, including metabolism, mitochondrial function, inflammation, and apoptosis. Reduced Sirt1 expression, in turn, exacerbates the loss of AMPK, causes an inflammatory response, including the transcription of NF-κB, and increases the formation of reactive oxygen species [47]. Also, Ruderman et al. said in a prior review that AMPK and SIRT-1 regulate each other and have several target molecules in common. Also, the clinical implications of these interactions, including their likelihood and their dysregulation, predispose to diseases such as type 2 diabetes and atherosclerotic cardiovascular disease and can be a therapeutic target [48]. Our current findings could be explained in the light of EMPA’s ability to reverse the induced reduction in hepatic AMPK/SIRT-1; in turn, the activation of NFκB is halted along with other cytokines; TNF-α and IL-6 [49,50,51].

The current investigation found that TAA induced an elevation of HIF-α. This result agrees with preceding studies on TAA-induced hepatic injury in which the HIF-α was the main culprit behind the derangement in liver tissue [52,53]. Interestingly, EMPA’s higher does counteract the induced elevation of hepatic HIF-α content. HIF has been implicated in the pathophysiology of various liver diseases [54]. Ryu et al. found that chronic activation of HIF-1α promoted apoptosis and fibrosis in cell-specific HIF-1α transgenic mice via the involvement of the SIRT-1 pathway [53].

A recent study implies that the antifibrotic effect of EMPA is related to its ability to suppress the Hippo pathway [55]. Another previous investigation studied the role of SGLT-2 inhibitor on NAFLD in leptin-deficient ob/ob mice and showed that SGLT-2 inhibitor action was independent of leptin [56]. Similarly, other outcomes were consistent in rodents models [57,58]. Another study indicated the possible action of SGLT-2 inhibitor on NAFLD is attributed to the regulation of endoplasmic reticulum (ER), oxidative stress, and autophagy. This regulation is manifested by the role of SGLT-2 inhibitor in controlling the level of proliferator-activated receptor-gamma coactivator-1 alpha (PGC-1α) levels and activation of the AMP-activated protein kinase (AMPK)/mammalian target of rapamycin (mTOR) signaling pathway [59,60,61]. Likewise, a meta-analysis clinical study showed that SGLT-2 Inhibitors dramatically lower hepatic enzyme levels retract hepatic fat, and enhance body composition [62]. Relatedly, clinical research correlated the action of SGLT-2 Inhibitors to their action on AMPK. Interestingly, the current study agreed with the aforementioned studies.

The limitation of the current study is that the proposed involvement of AMPK and HIF-1α and SIRT-1 should be emphasized by the use of knock-out models or the combination of suitable pharmacological blockers.

## 5. Conclusions

Collectively, for the first time to our knowledge, our current investigation showed the beneficial effect of EMPA in curbing TAA-induced hepatic fibrotic derangements. This effect was due to the stimulation of AMPK/ SIRT-1 and deterring the ROS, HIF-1α, inflammatory, and apoptotic marker levels.

## Figures and Tables

**Table 1:** Effect of empagliflozin on serum hepatic function in thioacetamide-intoxicated rats.

Groups	Albumin (g/dL)	GGT (nmol/mL)	ALT (U/L)	AST (U/L)	Ammonia (μg/mL)
Normal control	4.38 ± 0.133	3.12 ± 0.105	35.68 ± 3.154	63.35 ± 3.389	0.8 ± 0.134
TAA	1.73 ± 0.141 *	28.02 ± 2.936 *	181.68 ± 3.052 *	218.3 ± 3.353 *	3.6 ± 0.249 *
Emp (3 mg/kg)	2.32 ± 0.138 *^@^	14.98 ± 1.337 *^@^	98.68 ± 3.718 *^@^	121.72 ± 7.958 *^@^	2.13 ± 0.092 *^@^
Emp (6 mg/kg)	3.8 ± 0.157 *^@#^	6.22 ± 0.674 ^@#^	58 ± 4.261 *^@#^	87.02 ± 4.661 *^@#^	1.13 ± 0.093 ^@#^

Data are presented as mean ± SE. * vs. normal control group, ^@^ vs. TAA group, ^#^ vs. Emp (3 mg/kg) at *p* < 0.05.

**Table 2:** Effect of empagliflozin on oxidative stress biomarkers and lipid profile in thioacetamide-intoxicated rats.

Groups	GSH (nmol/mg Protein)	ATP (pg/mg Protein)	AMP (pg/mg Protein)	MDA (nmol/mg Protein)	Triglycerides (mg/dL)	Total Cholesterol (nmol/mL)
Normal control	1.7 ± 0.058	135.72 ± 8.765	215.78 ± 8.913	0.33 ± 0.026	87.68 ± 7.27	1.24 ± 0.078
TAA	0.48 ± 0.017 *	35.15 ± 3.338 *	72.23 ± 3.428 *	1.88 ± 0.02 *	185.3 ± 6.276 *	3.48 ± 0.117 *
Emp (3 mg/kg)	0.87 ± 0.042 *^@^	67.68 ± 2.272 *^@^	138.7 ± 8.108 *^@^	1.22 ± 0.043 *^@^	157.3 ± 4.157 *^@^	2.37 ± 0.046 *^@^
Emp (6 mg/kg)	1.65 ± 0.034 ^@#^	124.48 ± 2.836 ^@#^	208.5 ± 6.399 ^@#^	0.47 ± 0.047 ^@#^	100 ± 7.189 ^@#^	1.38 ± 0.053 ^@#^

Data are presented as mean ± SE. * vs. normal control group, ^@^ vs. TAA group, ^#^ vs. Emp (3 mg/kg) at *p* < 0.05.

## Data Availability

The data presented in this study are available upon request.

## Supplementary Materials

The following supporting information can be downloaded at: https://docs.google.com/document/d/1NTdUh9mfnE7yhvP0YVtbbUDgQ_L2G11s/edit.
